# Children’s Attraction to Physical Activity and Its Relation to Physical Activity Level

**DOI:** 10.21315/mjms2023.30.6.11

**Published:** 2023-12-19

**Authors:** Suet Kei Wu, Jyh Eiin Wong, Bee Koon Poh

**Affiliations:** Nutritional Sciences Programme and Centre for Community Health Studies (ReaCH), Faculty of Health Sciences, Universiti Kebangsaan Malaysia, Kuala Lumpur, Malaysia

**Keywords:** children’s attraction to physical activity (CAPA), deuterium dilution technique, physical activity, psychosocial factors, school children

## Abstract

**Background:**

Understanding of psychosocial factors of physical activity (PA) in children is crucial in encouraging sustained PA, which in turn is associated with important health outcomes. This study aimed to examine how children’s attraction to physical activity (CAPA) is associated with PA.

**Methods:**

This cross-sectional study was conducted among 219 primary school children (105 boys; 114 girls) aged 7 years old–10 years old in Kuala Lumpur, Malaysia in 2016–2017. Children from three main ethnicities, namely Malay, Chinese and Indian, were recruited. Weight, height and waist circumference were measured; body composition was assessed by deuterium dilution technique. CAPA and level of PA were obtained through self-administered questionnaires and reported as CAPA and PA scores.

**Results:**

Median CAPA and PA scores were 3.40 (Q_1_ = 3.00, Q_3_ = 3.80) and 2.31 (Q_1_ = 1.95, Q_3_ = 2.74), respectively. Significant gender differences were found in CAPA and PA scores, with boys being more attracted to PA (3.16 [Q_1_ = 2.90, Q_3_ = 3.44]; *P* = 0.001) and more physically active compared with girls (2.47 [Q_1_ = 2.07, Q_3_ = 3.07]; *P* = 0.001). CAPA and PA scores correlated positively in both sexes. Boys scored higher than girls in ‘liking of games and sports’ (ρ = 0.301, *P* = 0.002) and ‘liking of vigorous PA’ (ρ = 0.227, *P* = 0.02) CAPA subscales, which also correlated positively with PA scores. Girls’ PA scores correlated with ‘peer acceptance in games and sports’ (ρ = 0.329, *P* < 0.001).

**Conclusion:**

Boys are more physically active and have higher attraction to PA compared with girls. Differences in PA scores between the sexes were related to gender differences in CAPA scores. Thus, attention should be given to gender differences in CAPA related psychosocial factors when planning interventions to promote PA among children.

## Introduction

The health benefits of PA for adults and children are well-known. In children, PA plays significant roles in physical growth and also in cognitive development as well as in their social, emotional and mental health (1–3).

Globally, males are reported to be more physically active than females. This gender-based disparity persists from childhood to adulthood. Several studies have reported similar findings in Malaysian children of all age groups (4), including preschoolers (5), primary schoolers (6) and adolescents (7). Moreover, a nationwide study has revealed that the majority of Malaysian children not only have low levels of PA level but that they are also sedentary, with screen times exceeding the maximum recommendations (8).

Many factors have been reported as barriers and facilitators that influence children’s participation in PA (9). Studies have shown that interest in sports and attraction to PA are factors that can lead to higher levels of PA. Gender is also a factor; a study found that boys enjoyed games and participation in sports more than girls when examining the psycho-social correlates of PA among children in Portugal (10). Peer influence and perceived physical competence are also significant predictors for participation in PA among children and adolescents in Singapore and Thailand (11, 12). Self-confidence in the performance of PA also helps explain the levels of PA among young adolescents in Malaysia (13). Furthermore, parenting style, parental role modelling and encouragement have also been reported to influence children’s attraction to PA (14).

PA in children can be considered in relation to intrinsic motivation. Indeed, intrinsic motivation can help fulfil basic psychological needs, such as autonomy, competence and enjoyment (15). An individual who is intrinsically motivated will persist and maximise efforts when faced with challenging activities, while experiencing interest and enjoyment that increase or sustain participation (16). Previous studies have suggested that such positive outcomes as increased participation in and adherence to PA among adolescents and adults are attributable to intrinsic motivation (14). In this regard, however, there are few studies on the psychosocial correlates of children’s participation in PA, especially in Malaysia.

The main focus of this present study is children’s attraction to physical activity (CAPA), that is, the intrinsic interest and desire of children to engage in PA (17). It uses a multi-dimensional approach incorporating affective and cognitive dimensions, to identify different aspects of attraction to PA for primary school children (18). Empirical research has shown that higher levels of attraction to PA are associated with increased engagement in PA (19). Gender, race and cultural differences in attraction to PA and consequently participation in PA have also been reported (20). Hence, it is important to evaluate attraction to PA in different cultural contexts. Therefore, this study aimed to examine, among Malaysian primary school children of different ethnic backgrounds, how attraction to PA is associated with level of PA.

## Methods

### Study Design and Sampling

The cross-sectional survey was conducted in 10 primary schools located in Kuala Lumpur, the capital of Malaysia. Using purposive sampling, children aged between 7 years old and 10 years old from the Malay, Chinese and Indian ethnic groups were recruited. These three main ethnic groups were purposively selected from three types of schools, namely: i) national schools, ii) Chinese national-type schools and iii) Tamil national-type schools, to represent Malaysia as a multi-ethnic and multi-religious country. Exclusion criteria included physical disability, those with acute disease and those currently on medical treatment. Parents provided written, informed consent while children provided verbal assent prior to data collection.

### Sample Size Calculation

Sample size calculation is based on correlation (point biserial model) *t*-test performed in statistical software, G*Power version 3.1.9.2 (Heinrich Heine University, Düsseldorf, Germany). Based on effect size = 0.24 (21), power = 0.95, alpha = 0.05, the total sample size required is 258, after including a 20% dropout rate.

### Participants

A total of 245 children aged from 7 years old to 10 years old completed both the CAPA questionnaire and Physical Activity for Older Children (PAQ-C) questionnaire through face-to-face interviews during data collection at school. After excluding those children who provided missing or invalid responses to the questions on either of the questionnaires (*n* = 24), a total of 219 children (105 boys; 114 girls) were included in the analysis.

### Questionnaires

The CAPA consists of five subscales: i) liking of games and sports; ii) liking of physical exertion and exercise; iii) liking of vigorous PA; iv) peer acceptance in games and sports and iv) importance of exercise. The CAPA questionnaire is comprised of 25 items, with each sub-scale consisting of five items. The modified and validated version of the CAPA questionnaire was used in this study (21). All items were scored in Likert format, ranging from 1 (low) to 4 (high). Based on the CAPA scores, we used the 25th percentile and 75th percentile as the cut-off to define low, moderate and high CAPA levels in this sample.

Overall level of PA was measured using PAQ-C, validated and adapted from Kowalski et al. (22), that consists of nine questions to assess daily PA over the previous 7 days. Each item was scored on a 5-point scale. The level of PA was reported based on PA score, which was calculated as the average score of all nine items; higher scores indicate higher levels of PA (23).

Forward and backward translations were done to prepare Malay, Chinese and Tamil versions of the CAPA and PAQ-C instruments. A pilot trial was conducted with 30 primary school children for each language version to ensure that the questionnaires were worded appropriately and could be easily understood by the children. Face validity was confirmed by the pilot group. All versions of the questionnaires demonstrated acceptable internal reliability, with Cronbach’s alpha values ranging from 0.85 to 0.90 for CAPA and 0.58 to 0.67 for PAQ-C.

### Sociodemographic, Nutritional Status and Body Composition Variables

Information on the children’s sociodemographic backgrounds, including age, sex and ethnicity, was obtained through a parent-administered questionnaire. The children were categorised into three main ethnic groups, namely: i) Malay, ii) Chinese and iii) Indian, based on self-identification.

Weight was measured to the nearest 0.1 kg using a digital weighing scale (SECA model 880, Hamburg, Germany). Height was measured with a stadiometer (SECA model 213, Hamburg, Germany) to the nearest 0.1 cm. Body mass index (BMI) was calculated by dividing the measured weight (kg) with the square of height (m^2^). Waist circumference (WC) was measured to the nearest 0.1 cm at the midpoint between the lowest palpable rib and iliac crest using Lufkin tape (Model W606PM, Apex Tool Group, Maryland, USA) following the World Health Organization standardised protocols (24).

The deuterium dilution technique was used to assess total body water (TBW), fat free mass (FFM), fat mass (FM) and body fat percent of the children. An accurately weighed dose (0.3 g/kg body weight) of deuterium oxide (99.8% purity, Sigma-Aldrich, United Kingdom) was administered orally to the children. Prior to dosing, the children provided a saliva sample (2 mL) (collected in a clean, sterile and dry tube) to determine the background or natural deuterium enrichment. The post-dose samples were collected at 3 h and 3.5 h after dosing. Saliva samples were stored at −20 °C until analysis. The deuterium enrichment (mg/kg) in the saliva was measured using a Fourier Transform Infrared Spectrometer (FTIR Model 4500 series, Agilent, USA). All of the saliva samples were measured in duplicates. A calibration curve prepared with known standards of deuterium and measured by FTIR was used to determine the deuterium concentration of the subjects.

TBW was calculated based on this International Atomic Energy Agency (IAEA) formula (25):


TBW (kg)=[D2O dose (mg)/H2 enrichment in saliva (mg/kg)]/1.041

A two-compartment model was used to divide body mass (or weight) into FM and FFM. FFM (kg) is the product of TBW and the Lohman’s hydration coefficient specific for sex and age (26). Subsequently, FM (kg) is calculated as the difference between weight and FFM and body fat percent is calculated as the percentage of FM divided by body weight.

### Statistical Analysis

Statistical analyses were carried out using IBM SPSS Statistics for Windows, version 22.0 (IBM Corp., Armonk, New York, USA). Descriptive statistics was used to analyse the children’s characteristics, CAPA and PA scores. Means and standard deviations were used to report anthropometric measurements (normally distributed data), while median and inter-quartile ranges (IQR) were used to report CAPA and PA scores (continuous skewed data). Independent *t*-test was used to analyse differences between the sexes, unless otherwise stated. Pearson’s chi-square test was used to test the association between PA categories and CAPA categories with sociodemographic characteristics. Spearman’s rho (ρ) was used to analyse the relationship between CAPA and PA scores because the CAPA and PA scores were non-normally distributed (highly skewed). The significance level was set at *P* < 0.05.

## Results

The present study reports the findings from a study of 219 children (105 boys; 114 girls) with mean age of 9.02 (1.11) years old. Descriptive results of the children’s anthropometric characteristics are shown in Table 1. Girls had significantly higher body fat percentage, while boys had higher FFM (*P* = 0.002) and TBW (*P* = 0.002). Among ethnic groups, Indians had the highest body fat percentage while Chinese had the lowest (*P* < 0.05). Some 29.5% of boys and 24.6% girls were overweight or obese (χ^2^ = 1.388, df = 2, *P* = 0.05), with the highest proportion found among Indians (35.1%), followed by Malays (28.4%) and Chinese (17.9%) (χ^2^ = 13.474, df = 4, *P* = 0.009).

Table 2 shows significant differences in CAPA and PA scores between the sexes and among the ethnic groups. Median CAPA and PA scores were 3.40 (IQR = 3.00, 3.80) and 2.31 (IQR = 1.95, 2.74), respectively. Compared to girls, boys had higher median PA scores (*P* = 0.001), CAPA scores (*P* = 0.001) and for two of its subscales: ‘liking of games and sports’ (*P* < 0.001) and ‘liking of vigorous PA’ (*P* < 0.001). The girls had significantly higher median score in the ‘peer acceptance in games and sports’ subscale of CAPA (*P* = 0.03) compared to boys. Indian children had higher median CAPA scores compared to Chinese children (*P* < 0.05). Compared with Malay and Indian children, Chinese children had the lowest PA scores (*P* = 0.022).

Table 3 shows the correlation between CAPA scores and its subscales with PA scores. CAPA scores were positively correlated with PA scores in both boys (r_s_ = 0.207, *P* = 0.034) and girls (r_s_ = 0.236, *P* = 0.012). For boys, the subscale with the highest correlation was ‘liking of games and sports’ (r_s_ = 0.301, *P* = 0.002), followed by ‘liking of vigorous PA’ (r_s_ = 0.227, *P* = 0.02). However, among girls, ‘peer acceptance in games and sports’ was the only subscale that correlated significantly with PA score (r_s_ = 0.329, *P* < 0.001). Among Malay children only, their PA scores showed significant correlation with CAPA scores: overall score (r_s_ = 0.376, p = 0.002) liking of games and sports (r_s_ = 0.339, *P* = 0.005), liking of vigorous PA (r_s_ = 0.339, *P* = 0.005) and importance of exercise (r_s_ = 0.277, *P* = 0.023).

Table 4 shows the contributing factors to children’s PA level. Ethnicity, sex and CAPA score significantly predict PA level: *F* (5, 213) = 10.516, *P* < 0.001. When the children’s CAPA scores are higher, their PA levels are also higher (*β* = 0.231, *P* < 0.01). The PA levels of Chinese children are lower (*β* = −0.279, *P* < 0.01) compared to Malay children, while the boys score higher (*β* = 0.225, *P* < 0.01) in PA compared to the girls.

## Discussion

The primary purpose of this study was to examine the level of PA and link it with CAPA among Malaysian school children aged 7 years old–10 years old. The main findings indicate that children’s PA levels were associated with all subscales of CAPA, except for the ‘importance of exercise’ sub-scale. Significant differences were found between the boys and girls in PA levels, CAPA levels and its sub-scales of ‘liking of games and sports’, ‘liking of vigorous PA’ and ‘peer acceptance in games and sports’.

We found that, consistent with previous studies (8, 10, 13), the boys in our study had higher PA levels than girls. This study also found that most boys were more attracted to PA compared with girls. It has been postulated that these gender-based differences are related to the psycho-social dimensions of PA involvement (20). Boys tend to like games and sports more compared to girls, which is in agreement with previous empirical studies on sports participation. Boys were found to play sports more frequently than girls, while most girls engaged in dancing, skipping and drawing (27, 28).

As observed in the present study, boys enjoyed games and sports more and also liked vigorous PA more than girls. Deaner et al. (27) had also shown that boys were more motivated to participate in sports and more interested in pursuing sports in a competitive way. This could be because boys perceive themselves as having more self-efficacy and were more efficacious, and thus they achieve more from competitive sport activities than girls. As a result, boys’ participation in moderate-vigorous PA, during school break-time, is also enhanced (28). O’Connor et al. (29) also supported the finding that boys like to be involved in more vigorous PA, instead of participating in creative activities and spontaneous sports, which are activities more favoured by girls. Furthermore, it has been reported that girls prefer fitness activities over competitive sports because they find fitness skills easier to learn than sports skills (30). In contrast, Seabra et al. (10) have reported that normal weight girls are attracted to vigorous PA. However, other research has indicated that children found PA more enjoyable when they are encouraged to experiment in different activities instead of being forced to compete and win (30).

Interestingly, the present study also revealed that ‘peer acceptance in games and sports’ is the only CAPA subscale that is significantly associated with girls’ level of PA. Similar to our findings, the participation of Portuguese girls in PA was also significantly and positively associated with perceived acceptance by peers in games and sports (10). Girls show positive emotion when they are with their peers, and hence, they are more active when they can share their activities with peers (31). Consequently, the presence of peers and friends who bring enjoyment and provide social support network is important to motivate participation in PA among girls (32). Furthermore, the differences in the levels of PA among boys and girls in the Malaysian primary school student population have been attributed to differences in sports socialisation (33). As certain activities are believed to be culturally inappropriate for girls due to the socialisation pattern, girls’ opportunities to be physically active are more limited (34).

Our study found that Indian children are relatively more active compared to Malay and Chinese children, while Chinese children achieve the lowest PA score. A nationally representative study of 7-year-old to-12-year-old Malaysian school children also reported that Chinese children achieved the lowest PA scores (6). However, that same study reported that Malay children scored the highest in PA level. In contrast, ethnic differences in PA participation were not observed among a Malaysian adolescent sample (13).

When we examined the association between attraction to PA and level of PA by ethnic groups, we found that significant associations were found only among Malay children. Specifically, ‘liking of games and sports’ as well as ‘liking of vigorous PA’ were associated with higher levels of PA among Malay children. However, Raj et al. (35) found that Malay children were more prone to engage in excessive screen time. Moreover, Lee et al. (8) reported that Malaysian boys regularly engage in sedentary behaviour despite having high level of PA. In contrast, Brustad determined that ethnicity was not a significant contributor to the differences in the patterns of attraction to PA among children in the United States (19). However, according to Lian et al. (36), when the same PA patterns were applied to Malaysian adults, the Chinese were the least active compared to other ethnicities. Lau et al. (37) revealed that, among Chinese children in Hong Kong, the parents’ overall PA orientation and their overweight child’s attraction to PA were positively correlated, although the association is relatively small. In fact, it was also indicated that paternal role modelling and enjoyment of activity and maternal encouragement of activity were correlated with overweight children’s attraction to PA. Hence, this may indicate that children’s participation in PA as well as their attraction to PA are actually multifaceted. Like all the past studies cited, the present findings provide additional insights into psycho-social correlates of PA that contribute to children’s participation in PA.

Our study has several limitations. The study design was cross-sectional, which limits any interpretation of a causal relationship between CAPA with their participation in PA. CAPA and PA were based on self-reporting by the children and their parents. Hence, the data may be prone to be certain forms of reporting bias, specifically parental bias, and present habits and perceptions. In spite of these limitations, to the best of our knowledge, this is the first study to examine the associations between CAPA and levels of PA among school children of different ethnicities in Malaysia. These findings are important to provide insights into the psychological and social aspects of children’s participation in PA, which in turn can aid in the development of strategies and interventions to promote PA among Malaysian children.

## Conclusion

In summary, the present findings demonstrate that boys are more physically active and appear to have higher attraction to PA than girls. This study also highlights that boys and girls differ in several aspects regarding their attraction to PA. Boys appear to like games and sports as well as vigorous PA compared with girls. These findings reiterate the importance of peer influence in the participation of PA, particularly among girls. Although Chinese children appear to be the least physically active compared with Malay and Indian children, there were no major ethnicity-based differences that were observed in the relationship between these CAPA and their level of PA. These findings suggest that gender differences in psychosocial aspects, specifically attraction to PA, should be taken into account when planning more effective intervention and promotion of PA to counteract the sedentary lifestyle that is responsible for the growing epidemic of overweight and obesity in Malaysian children.

## Figures and Tables

**Table 1:** Anthropometric characteristics of children by sex and ethnicity (mean ± SD)

	Sex				Ethnicity			
	
	All (*N* = 219)	Boys (*n* = 105)	Girls (*n* = 114)	Chi-square; *P*-value	Malays (*n* = 67)	Chinese (*n* = 78)	Indians (*n* = 74)	Chi-square test; *P*-value
Age (years old)	9.02 ± 1.11	9.06 ± 1.11	8.97 ± 1.13		9.06 ± 1.03	9.00 ± 1.18	9.00 ± 1.14	
Weight (kg)	30.1 ± 9.2	31.7 ± 9.4	30.8 ± 9.7		30.4 ± 10.0	28.9 ± 8.2	31.2 ± 9.5	
Height (cm)	131.9 ± 8.8	134.3 ± 8.4	133.0 ± 9.3		130.4 ± 8.0	131.5 ± 9.3	133.6 ± 8.8	
BMI (kg/m^2^)	17.04 ± 3.59	17.27 ± 3.71	16.82 ± 3.46		17.54 ± 4.13	16.44 ± 2.91	17.21 ± 3.66	
BMI status				χ^2^ = 1.388; *P* = 0.050				χ^2^ = 13.474; *P* = 0.009†
Underweight (*n*, %)	18 (8.2)	10 (9.5)	8 (7.0)		7 (10.4)	2 (2.6)	9 (12.2)	
Normal weight (*n*, %)	142 (64.8)	64 (61.0)	78 (68.4)		41 (61.2)	62 (79.5)	39 (52.7)	
Overweight/obese (*n*, %)	59 (26.9)	31 (29.5)	28 (24.6)		19 (28.4)	14 (17.9)	26 (35.1)	
Waist circumference (cm)	58.2 ± 10.0	60.2 ± 10.6	58.4 ± 9.8		58.9 ± 11.07	56.7 ± 8.6	59.2 ± 10.16	
Body fat (%)	31.8 ± 7.9	30.8 ± 8.5	32.8 ± 7.2*		30.8 ± 8.8^ab^	30.7 ± 6.9^a^	33.9 ± 7.8^b^	
FM (kg)	10.1 ± 5.6	10.2 ± 6.1	10.0 ± 5.1		10.0 ± 6.1	9.3 ± 4.9	11.1 ± 5.7	
FFM (kg)	20.0 ± 7.9	20.8 ± 4.0*	19.3 ± 4.4		20.3 ± 4.5	19.6 ± 3.9	20.1 ± 4.4	
TBW (kg)	15.4 ± 3.2	16.0 ± 3.1*	14.8 ± 3.3		15.6 ± 3.4	15.0 ± 2.9	15.4 ± 3.4	

Notes:

* Significant difference between the sexes at *P* < 0.05 using Mann-Whitney U test;

† Significant difference among ethnicities at *P* < 0.05 using chi-square test;

ab Different superscript letters show significant differences by ethnicities at *P* < 0.05 using Kruskal-Wallis and Bonferroni post-hoc tests

**Table 2:** PA score, CAPA subscales and overall scores of children [median (Q_1_, Q_3_)]

	PA score	*P*-value	Liking of games and sports	*P*-value	Liking of physical exertion	*P*-value	Liking of vigorous PA	*P*-value	Peer acceptance in games and sports	*P*-value	Importance of exercise	*P*-value	CAPA score	*P*-value
All participants (*N* = 219)	2.31 (1.95, 2.74)		3.00 (2.80, 3.40)		3.40 (2.80, 3.80)		2.40 (2.00, 2.60)		3.40 (3.00, 3.80)		3.08 (2.76, 3.32)		3.40 (3.00, 3.80)	
Sex														
Boys (*n* = 105)	2.47** (2.07, 3.01)	0.001	3.60** (3.25, 3.80)	< 0.001	3.20 (2.80, 3.60)	0.008	3.40** (3.00, 3.80)	< 0.001	2.30 (2.05, 2.80)	0.03	3.40 (3.00, 3.80)	0.824	3.16* (2.90, 3.44)	0.001
Girls (*n* = 114)	2.19 (1.86, 2.57)		3.00 (2.80, 3.20)		3.20 (2.60,3.60)		2.40 (2.00, 2.60)		3.40* (2.80, 3.60)		3.04 (2.76, 3.20)		3.00 (2.80, 3.60)	
Ethnicity														
Malays (*n* = 67)	2.46 a (2.08, 2.79)	< 0.001	3.40 (3.00, 3.80)	0.415	3.00 (2.80, 3.40)	0.162	3.40 a (2.80, 3.80)	0.015	2.40 a (2.00, 2.60)	< 0.001	3.40 (3.00, 3.80)	0.419	3.08 ab (2.76, 3.32)	0.022
Chinese (*n* = 78)	2.04 b (1.73, 2.42)		3.40 (3.00, 3.80)		3.00 (2.60, 3.40)		3.00 b (2.60, 3.45)		2.20 a (2.00, 2.60)		3.40 (3.00, 3.80)		3.00 a (2.76, 3.25)	
Indians (*n* = 74)	2.57 a (2.19, 2.98)		3.50 (3.00, 3.80)		3.20 (2.60, 3.60)		3.20 a (2.60, 3.60)		2.80 b (2.20, 3.00)		3.60 (3.00, 4.00)		3.20 b (2.88, 3.48)	

Notes:

* Significant difference between the sexes using Mann-Whitney U test at:

* *P* < 0.05;

** *P* < 0.01;

ab Different superscript letters show significant differences between ethnicities using Kruskal-Wallis and Dunn-Bonferroni post-hoc tests at *P* < 0.05;

PA = physical activity; CAPA = children’s attraction to physical activity

**Table 3:** Spearman’s correlation (r_s_) between CAPA and PA scores among children (*N* = 219)

	Liking of games and sports	*P*-value	Liking of physical exertion	*P*-value	Liking of vigorous PA	*P*-value	Peer acceptance in games and sports	*P*-value	Importance of exercise	*P*-value	CAPA score	*P*-value
All participants (*N* = 219)	0.221	0.001*	0.136	0.044*	0.236	< 0.001**	0.257	< 0.001**	0.074	0.275	0.258	< 0.001**
Sex												
Boys (*n* = 105)	0.301	0.002**	0.062	0.527	0.227	0.020*	0.106	0.281	0.075	0.447	0.207	0.034*
Girls (*n* = 114)	0.075	0.428	0.158	0.093	0.149	0.113	0.329	< 0.001**	0.089	0.348	0.236	0.012*
Ethnicity												
Malays (*n* = 67)	0.339	0.005*	0.189	0.126	0.339	0.005*	0.236	0.055	0.277	0.023*	0.376	0.002**
Chinese (*n* = 78)	0.182	0.111	0.205	0.072	0.161	0.158	0.201	0.077	−0.044	0.700	0.189	0.098
Indians (*n* = 74)	0.035	0.764	−0.171	0.145	−0.025	0.829	0.096	0.416	−0.026	0.827	−0.023	0.845

Notes: Spearman’s rho significant at :

* *P* < 0.05;

** *P* < 0.01;

PA = physical activity; CAPA = children’s attraction to physical activity

**Table 4:** Contributing factors to children’s PA level

Model	Variables	F	*R* ^2^	Adjusted *R*^2^	β	*P*-value
		10.327	0.195	0.176		< 0.001***
1	Liking of games and sports				0.159	0.014*
	Male				0.226	0.002**
	Chinese				−0.295	0.001**
	Indians				0.161	0.072
		9.072	0.176	0.156		< 0.001***
2	Liking of physical exertion				0.061	0.324
	Male				0.262	< 0.001***
	Chinese				−0.285	0.002**
	Indians				0.177	0.050
		10.128	0.192	0.173		< 0.001***
3	Liking of vigorous PA				0.146	0.022*
	Male				0.222	0.003**
	Chinese				−0.259	0.004**
	Indians				0.177	0.048*
		9.843	0.188	0.169		< 0.001***
4	Peer acceptance in games and sports				0.145	0.042*
	Male				0.248	0.001**
	Chinese				−0.289	0.001**
	Indians				0.131	0.158
		9.671	0.185	0.166		< 0.001***
5	Importance of exercise				0.119	0.065
	Male				0.269	< 0.001***
	Chinese				−0.304	0.001**
	Indians				0.165	0.067
		10.516	0.198	0.179		< 0.001***
6	CAPA score				0.231	0.009*
	Male				0.225	0.002**
	Chinese				−0.279	0.002**
	Indians				0.150	0.094

Notes: Reference groups are female and Malays, for sex and ethnicity, respectively and adjusted with age; Multiple regression test:

* *P* < 0.05,

** *P* < 0.01,

*** *P* < 0.001
